# A Review of the Pedunculopontine Nucleus in Parkinson's Disease

**DOI:** 10.3389/fnagi.2018.00099

**Published:** 2018-04-26

**Authors:** Isobel T. French, Kalai A. Muthusamy

**Affiliations:** Division of Neurosurgery, Department of Surgery, University Malaya, Kuala Lumpur, Malaysia

**Keywords:** Pedunculopontine nucleus, mesenphalic locomotor region, basal ganglia, substantia nigra, brainstem, Parkinson's disease, deep brain stimulation

## Abstract

The pedunculopontine nucleus (PPN) is situated in the upper pons in the dorsolateral portion of the ponto-mesencephalic tegmentum. Its main mass is positioned at the trochlear nucleus level, and is part of the mesenphalic locomotor region (MLR) in the upper brainstem. The human PPN is divided into two subnuclei, the pars compacta (PPNc) and pars dissipatus (PPNd), and constitutes both cholinergic and non-cholinergic neurons with afferent and efferent projections to the cerebral cortex, thalamus, basal ganglia (BG), cerebellum, and spinal cord. The BG controls locomotion and posture via GABAergic output of the substantia nigra pars reticulate (SNr). In PD patients, GABAergic BG output levels are abnormally increased, and gait disturbances are produced via abnormal increases in SNr-induced inhibition of the MLR. Since the PPN is vastly connected with the BG and the brainstem, dysfunction within these systems lead to advanced symptomatic progression in Parkinson's disease (PD), including sleep and cognitive issues. To date, the best treatment is to perform deep brain stimulation (DBS) on PD patients as outcomes have shown positive effects in ameliorating the debilitating symptoms of this disease by treating pathological circuitries within the parkinsonian brain. It is therefore important to address the challenges and develop this procedure to improve the quality of life of PD patients.

## The pedunculopontine nucleus

The pedunculopontine nucleus (PPN) is situated in the upper pons in the dorsolateral part of the ponto-mesencephalic tegmentum. Its main mass is located at the level of trochlear nucleus, and is part of the mesenphalic locomotor region (MLR) in the upper brainstem (Olszewski and Baxter, 1954; Geula et al., 1993). Olszewski and Baxter (1954) divided the human PPN into two subnuclei, the pars compacta (PPNc) and pars dissipatus (PPNd). The PPNc is more prominent with a compact cluster of large neurons, whereas the PPNd is composed of small and medium-sized neurons scattered inside the superior cerebellar peduncle (SCP) and central tegmental tract (Olszewski and Baxter, 1954). The PPN comprises both cholinergic and non-cholinergic neurons, and possesses afferent and efferent projections to the cerebral cortex, thalamus, basal ganglia (BG), cerebellum, and spinal cord.

Eighty to ninety percentage of the PPNc contains cholinergic neurons amassed along the dorsolateral border of the SCP at trochlear nucleus levels with few dopaminergic neurons (Jones, 1991; Pahapill and Lozano, 2000; Winn, 2008). These thin unmyelinated axons diverge extensively over the brain supply nuclei in the BG, cerebellum, reticular formation in the lower brainstem, and also the spinal cord (Stein, 2009). PPNd neurons dispersed along the SCP from mid-encephalic to mid-pontine levels constitute mainly glutamatergic neurons (Rye et al., 1987; Lavoie and Parent, 1994a) while the rest are cholinergic (Mesulam et al., 1989).

**Figure 1:**
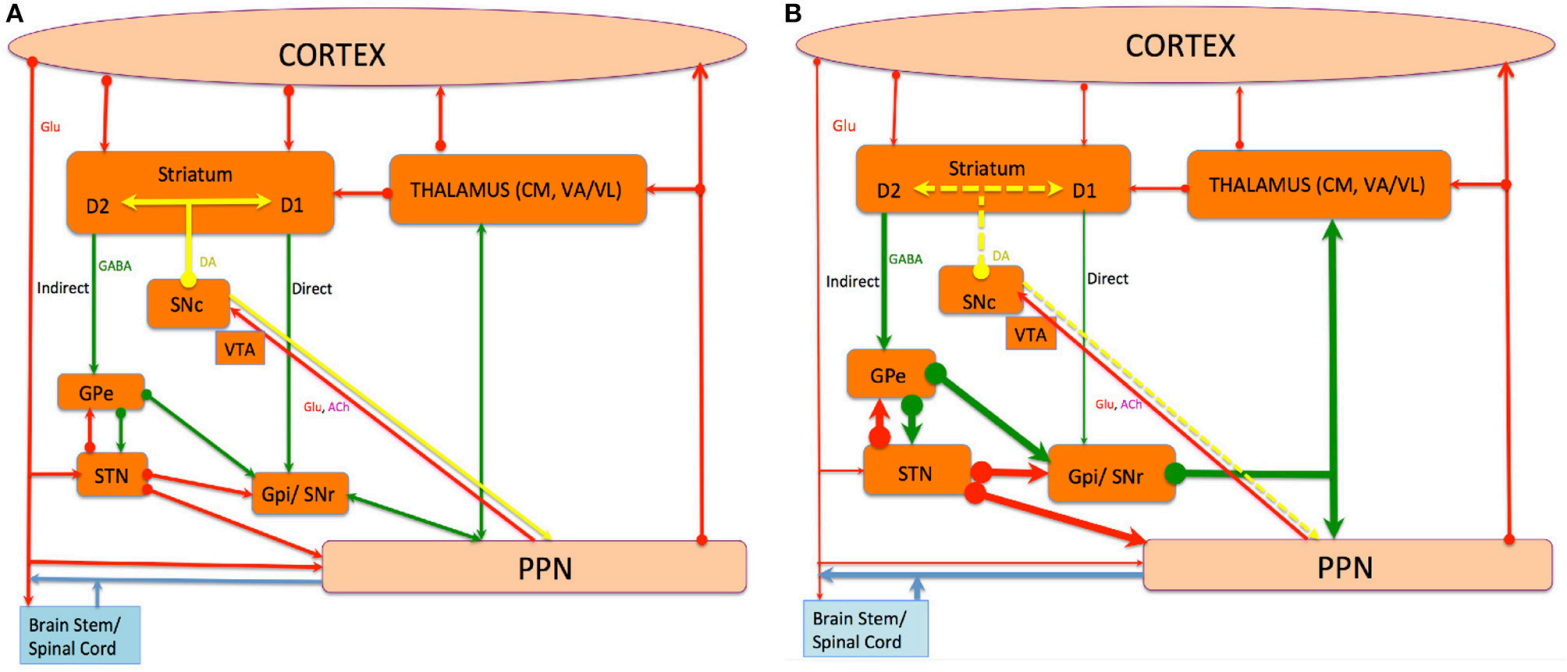
The connections of The PPN, and the direct and indirect pathway of BG-thalamocortical circuits under normal **(A)** and PD **(B)** conditions. Red, green, and yellow lines denote glutamatergic, GABAergic and dopaminergic projections respectively, while blue lines indicate chemically amalgamated projections. Thickening lines show increased activity whereas thinning lines show decreased activity when alterations occur in the average activity rate of specific projection pathways in PD compared with the normal state. Dotted yellow lines indicate loss. The striatum and STN deliver input from incoming cortical information to the BG. The GPi and SNr deliver output information from the BG to the rest of the brain and apply robust inhibitory control on targets in the thalamus and the brainstem. This tonic inhibitory input must be disinhibited to permit normal movements to occur. The striatum applies opposite influences on the GPi and SNr via two distinct classes of efferent neurons, namely the D1-receptor-rich “direct pathway” positively modulated by DA and the D2-receptor-rich “indirect pathway” negatively modulated by DA. The loss of DA in PD's causes disequilibrium in the activity of these two striatofugal pathways and their corresponding cortical inputs.

The PPNc and PPNd also possess GABAergic inhibitory neurons, whereas cholinergic neurons also contain neuropeptides and novel neuromodulators (Vincent et al., 1986; Vincent and Kimura, 1992; Lavoie and Parent, 1994a,b,c; Bevan and Bolam, 1995). The PPN possesses ascending and descending afferent and efferent (see Figure 1) projections, and PPN inputs approach from above and below its level. Descending networks from the cerebral cortex project via the BG and extrapyramidal system to the PPN, including the face, arm, trunk, and leg areas of the motor cortex (MCx), specifically Brodmann area 4 (von Monakow et al., 1979). The PPNc is also a primary constituent in a feedback loop to the thalamus from the spinal cord and limbic system (Pahapill and Lozano, 2000) and is a component of the ascending reticular activating system (ARAS), where cortical stimulation is modulated via ascending cholinergic connections to the thalamus (Steriade, 2004).

The PPN enhances the movement, motivational, and cognitive aspects of multifaceted behavioral responses (Garcia-Rill, 1986; Inglis and Winn, 1995; Reese et al., 1995; Takakusaki et al., 2004a). Stimulation of this area induces locomotion in animals, whereas damage leads to a number of neurological disorders included in Parkinson's disease (PD), Alzheimer's disease and schizophrenia due to its close ties with the BG and thalamus.

### PPN connectivity and physiology

#### Motor cortical connections

The PPN possesses dense connections to the upper extremity regions of the MCx, followed by the lower extremity, trunk, and orofacial regions. Connections are more dense in the pre-MCx and frontal lobe compared to other regions (Muthusamy et al., 2007). Reciprocal connections also exist with the ipsilateral prefrontal MCx (Pahapill and Lozano, 2000). The PPN also obtains direct cortical afferent fibers from the primary and somatosensory motor area, pre-supplementary, dorsal, and ventral pre-MCx, as well as frontal eye fields (Kuypers and Lawrence, 1967; Moonedley and Graybiel, 1980; Matsumura et al., 2000). These connections implicate the PPN in cortical functions such as movement, cognition and sleep.

#### Striatal connections

PPN efferent projections contacting the striatum are poorly arborized, excluding the ventral and peri-pallidal zone of the putamen (Lavoie and Parent, 1994c). These connections indicate that the PPN is also involved in limbic function. This is seen in the ventral striatum, also known as the nucleus accumbens (NAcc). Ascending PPN connections provide control over striatal input and output via connections with the thalamus and cerebral cortex, even in the absence of direct projections (Winn, 1998). PPN stimulation increases bursting activity in the NAcc (Floresco et al., 2003), where changes in release accompanying different firing patterns reveal two forceful conditions in dopamine (DA) levels in the striatum and NAcc. This is namely a tonic state with truncated but stable DA levels, and a phasic state correlated to behavioral actions and reactions to environmental stimuli. PPN cholinergic inputs therefore provide a functional duality ensuring the basal level of DA neurons response, whether stimulus-specific or anatomically diffuse. This also determines the response required in precise circumstances (Mena-Segovia et al., 2008b).

#### Thalamic connections

Ascending PPN outputs project via the ventral and dorsal tegmental bundle pathways carry major cholinergic projections (Garcia-Rill, 1991) to all thalamic nuclei (Lavoie and Parent, 1994b). Strong cholinergic innervations to the intralaminar and reticular nuclei were also revealed (Mesulam et al., 1992a). These studies suggest that the thalamus obtains major cholinergic PPN input, especially toward “nonspecific” nuclei associated with the ARAS. The ARAS (Moruzzi and Magoun, 1949) stimulates the cortex using cholinergic input to the thalamus largely via PPN cholinergic cells (Pare et al., 1988; Steriade et al., 1988). This projection then travels to non-specific thalamic nuclei and produces rapid cortical oscillatory activity associated with arousal and rapid eye movement sleep (REMS) (Steriade, 2004). This stimulates reticular formation neurons in a positive-feedback procedure, whereas termination is induced through inhibitory activity of REMS-off aminergic neurons via REMS-on stimulated neuronal regulation positioned in the laterodorsal tegmental nucleus (LDT), and PPN regions (French and Muthusamy, 2016).

#### Pallidal connections

The globus pallidus interna (GPi) of the globus pallidum (GP) sends inhibitory efferent fibers to the ipsilateral PPN. Anterograde tracer studies reveal that the PPN sends substantial efferent fibers to the GPi (Lavoie and Parent, 1994b) rather than the globus pallidus externa (GPe). In humans, the GP receives cholinergic innervations from the brainstem (Mesulam et al., 1983). Pallidal efferent pathways descend along the pallidotegmental tract to the Forel's field before dividing into the medial & lateral descending pathway into the PPN and midbrain tegmentum. The medial pathway then joins the medial longitudinal fasciculus in the pre-rubral field & terminates in the PPN, whereas the lateral pathway descends in the ventrolateral tegmentum before intermingling with the medial lemniscus and terminating in the PPN (Carpenter, 1976; DeVito et al., 1980).

#### Nigral connections

Afferent gamma aminobutyric acid (GABA) endings from the substantia nigra (SN) profusely contact with synapses of PPN cell bodies and dendrites (Granata and Kitai, 1991). Reciprocally, the PPN sends efferent glutamatergic and cholinergic fibers to dopaminergic SN pars compacta (SNc) neurons (Charara et al., 1996) via multiple contacts with dendrites and cell bodies (Bolam et al., 1991; Mesulam et al., 1992b; Charara et al., 1996). These connections propose that strong excitatory influences on dopaminergic SNc neurons exerted from the PPN as pedunculonigral fibers branch more profusely in the SNc than SN pars reticulata (SNr).

The PPN also receives DA innervation over its anteroposterior extent (Rolland et al., 2009) from the SNc at posterior and mediodorsal levels, crossing through the medial lemniscus and reticular formation. These fibers tend to avoid cholinergic cell bodies but converge in neighboring non-cholinergic PPN parts through an anteroposterior and ventrodorsal gradient, particularly in the ventral cuneiform nucleus (CuN) located dorsally to the PPN. Furthermore, cell bodies analogous to the dopaminergic peri- and retrorubral cell clusters decrease rapidly posteriorly in the anterior PPN. Lavoie and Parent (1994b) also report that DA and cholinergic cells dominate adjoining but definite regions, with the dopaminergic population more anteriorly and laterodorsally located. Thus, the PPN along with the CuN receives dopaminergic innervation, endorsing that DA has a role in neural activity modulation of these structures. Intriguingly, DA fibers are heterogeneously dispersed, with central concentrations in the non-cholinergic PPN and ventral CuN border. This implies that functions such as postural muscle tone controlled by the PPN or locomotion via the CuN (Takakusaki et al., 2003) are directly influenced by DA. Furthermore, PPN and nigral dopaminergic neurons ascertain a direct loop moderating motor activity as both the cholinergic and especially non-cholinergic PPN project back to dopaminergic neurons of the SNc and ventral tegmental area (VTA) (Lavoie and Parent, 1994b; Mena-Segovia et al., 2008a).

PPN cholinergic projections have an expansive effect upon midbrain DA systems innervating both SNc and VTA neurons. Though less significant in controlling burst firing and population action of DA neurons, PPN neurons could be associated with sustaining the muscarinic-dependent tonic discharge of DA and specifying DA neuronal phasic signals to time sensory events. This suggests a responsibility for the PPN in associative learning. These phasic signals most likely work as a part of the ARAS in contribution of acetylcholine (ACh), to thalamocortical neuronal coherence in sensory stimuli integration.

PPN connections to the SNc and VTA alters DA release in different regions of the striatum, further affecting striatal inputs such as the cortex and thalamus. This modifies activity throughout the BG that eventually leads to behavioral changes. PPN afferents increase the number of neuronal burst firing in the VTA, though only in neurons that are already firing (Floresco et al., 2003). Extensive bilateral cholinergic innervation is also observed in the VTA, deriving primarily from the LDT and caudal PPN. An ipsilateral cholinergic projection originating from less dense regions of the cholinergic group projects to the SN. Cholinergic and glutamatergic PPN cells projecting to the SN and VTA (Beninato and Spencer, 1987; Bolam et al., 1991; Futami et al., 1995). This activates midbrain DA cells with short latencies (Scarnati et al., 1984; Lokwan et al., 1999) and evokes DA release in dopaminergic innervation areas (Forster and Blaha, 2003). Such topography indicates that cholinergic outflow from the PPN to functionally different systems vary depending on where afferent input is received. Input to dense cores of the group appears to affect cholinergic outflow to the mesolimbic DA system rather than the nigrostriatal system. However, this does not negate its influence on the nigrostriatal system as SN-projecting cells are also found throughout areas containing cholinergic cells. This implies that input received by an SN-projecting cell is more likely to affect the ipsilateral nigrostriatal system rather than the contralateral side. The VTA, however, appears to receive input from both sides of the cholinergic group. The identification of a distinct difference in cholinergic innervation of the SN and VTA relays important information on how cholinergic systems regulate CNS-controlling behavioral states including arousal and motor functions (Steriade and Buzsaki, 1990). Relative to this, the PPN also works with a parallel cholinergic input arising from the LDT. The PPN is thus part of two interrelated systems arising from cholinergic brainstem neurons modulating DA systems in the midbrain, another of which is the LDT.

#### Subthalamic nuclei connections

Glutamatergic afferents from the subthalamic nucleus (STN) to the PPN function through a positive feed-forward circuit arising from PPN cholinergic neurons. These projections converge with inputs from the cortex and GPe, affecting the activity of direct and indirect pathways (Bevan and Bolam, 1995).

A subpopulation of PPN neurons with ascending projections to the STN are distinct from neurons with descending projections to the gigantocellular reticular nucleus (GiN) (Mena-Segovia et al., 2008a; Ros et al., 2010). PPN projections are predominantly discrete to these two motor components, although cholinergic and non-cholinergic projections also surface from neurons within similar areas (Mena-Segovia et al., 2008a; Ros et al., 2010). This suggests that projection neurons in both pathways interact with each other, advocating an integrative role within PPN microcircuits. Similarly, the distribution of cholinergic, GABAergic, and glutamatergic neurons (Mena-Segovia et al., 2009; Wang and Morales, 2009; Martinez-Gonzalez et al., 2012) suggests that the rostral PPN is chiefly inhibitory being GABAergic, while the caudal PPN is chiefly excitatory being glutamatergic. Hence, motor projections to the STN and GiN are primarily glutamatergic with distinctive subtypes as they contain a diverse balance of calcium-binding proteins. Nonetheless, GABAergic constituents also exist (Bevan and Bolam, 1995). The quantity of cholinergic neurons in the caudal PPN is larger connecting to both targets, suggesting that cholinergic-mediated excitation of motor structures arise from the caudal PPN. Descending non-cholinergic neurons showed distinct electrophysiological properties compared to ascending non-cholinergic neurons, supporting the existence of functional differentiations concerning these two routes (Ros et al., 2010). Thus, descending PPN projections mediated via reticulospinal neurons of the GiN excites inhibitory interneurons in the spinal cord and modulate excitatory MLR output (Takakusaki et al., 2004a; Takakusaki, 2008). This concurs that the PPN is implicated in locomotion initiation (see Figure 1A).

#### Cerebellar and spinal cord connections

Efferent fibers from deep cerebellar nuclei send collaterals to the PPN before reaching the thalamus (Hazrati and Parent, 1992), suggesting that the PPN acts as a well-designed consolidation epicenter between the BG and the cerebellum. Matsumura et al. (1997) also suggests that the PPN acts as a dispatch between the cerebral cortex and spinal cord, performing as a brainstem regulator center for interlimb movement synchronization and bimanual motor performance (Matsumura et al., 1997).

## Parkinson's disease

PD is a collection of neurodegenerative conditions affecting the brain, particularly pigmented nuclei in the extrapyramidal system of the midbrain and brainstem, the olfactory tubercle, cerebral cortex, and components of the peripheral nervous system (Braak et al., 2003). Ultimate physical debilities ensuing from these pathologies are motor deficiencies termed “parkinsonism.” These comprise dearth and movement slowness, known as akinesia and bradykinesia, muscle rigidity and resting tremor. Parkinsonism is produced primarily through BG functional impairments.

Principally, these problems result from dopaminergic neuronal degeneration in the midbrain leading to DA deficiency in areas receiving dopaminergic inputs, specifically from the post-commissural putamen and other BG areas (Braak et al., 2003). However, before dopaminergic degeneration occurs in the midbrain, Lewy neurites (LNs), and bodies (LBs), first form in the non-catecholaminergic dorsal glossopharyngeus-vagus complex and intermediate reticular zone projection neurons, and exclusive gain setting system nerve cell types, which are the coeruleus-subcoeruleus complex, caudal raphe nuclei, GiN, and olfactory bulb, tract, and/or anterior nucleus before nigral involvement (Del Tredici et al., 2002). This is possibly why PD patients develop anosmia during initial stages. This multisystem disorder first involves few susceptible nerve cell types in particular areas of the human nervous system, where the intracerebral development of abnormal proteinaceous LBs and LNs commences at definite locations and progress in a topographic order (Braak and Del Tredici, 2004). As the disease advances, constituents of the autonomic, limbic, and somatomotor systems become increasingly compromised. During pre-symptomatic stages 1–2, LB inclusion pathology is constricted to the medulla oblongata/pontine tegmentum and olfactory bulb/anterior nucleus. This means that SN involvement presumes an obvious prevailing pathology in the medulla oblongata. If it were possible to detect PD during this stage with an underlying therapy available, consequent neuronal loss in the SN could probably be prevented (Braak et al., 2003). In stages 3–4, the SN and other midbrain gray nuclei and forebrain undergo severe pathological changes as the process develops in an ascending manner traversing the upper border of the pontine tegmentum and enters midbrain and forebrain basal portions. More explicitly, the very first solitary LNs are observed in the SNc leading to granular aggregations, pale bodies, and LBs in melanized projection neurons, all of which are thin and sparsely myelinated axons (Braak et al., 2004). Classically, nigral pathology initiates in the SNc postero-lateral subnucleus (Braak and Braak, 1985; Gibb and Lees, 1991) and continue on in the postero-superior and posteromedial subnuclei, circumventing the SN magnocellular and anterior subnuclei while causing trivial lesions (Braak et al., 2003). Nuclear gray pathology from earlier stages is now severely exacerbated. Concurrently, the process impinges on the central amygdala subnucleus before extending into basolateral nuclei. LN complexes progressively fill the central subnucleus and characterize it off from contiguous structures (Sims and Williams, 1990; Amaral et al., 1992; Braak et al., 1994; Bohus et al., 1996). Other brain regions involved include the cholinergic PPN (Garcia-Rill, 1991; Inglis and Winn, 1995; Rye, 1997; Pahapill and Lozano, 2000), oral raphe nuclei, cholinergic magnocellular nuclei of the basal forebrain (Candy et al., 1983; Whitehouse et al., 1983; Mesulam et al., 1992a), and hypothalamic tuberomamillary nucleus (Del Tredici and Braak, 2004).

Excluding the SN and PPN, other striatal loop axes begin early myelination and oppose undergoing pathological changes (Braak et al., 2004). At stage 4, the poorly myelinated temporal mesocortex involving the transentorhinal region between the allocortex and neocortex is engaged in disease development for the first time (Braak et al., 2003). Most patients transcend into the symptomatic stages at this juncture. In the last stages 5–6, the disease reveals itself in all of its clinical dimensions as the process crosses the mature neocortex (Braak et al., 2004). During this stage, a plexus of LNs develop in the second sector of Ammon's horn (Dickson et al., 1994). This feature is typical of stages 4–6 that even when sections through the SN are unavailable, PD can be diagnosed based on Ammon's horn lesions alone (Del Tredici and Braak, 2004). During these final stages, the neurodegenerative progression reaches its supreme topographic degree. The SN appears practically stripped of melanoneurons, appearing colorless upon macroscopic inspection (Braak et al., 2004).

## The BG and PD

The BG comprises the neostriatum containing the caudate nucleus (CN) and putamen, the GP containing the GPe and GPi, the STN, and the SN consisting of the SNr and SNc. These structures contribute to anatomically and functionally isolated loops involving certain thalamic and cortical regions. These parallel circuits differ based on the cortical function involved and are separated into “motor,” “associative,” and “limbic” loops (Alexander et al., 1986; Alexander and Crutcher, 1990; Middleton and Strick, 2000; Kelly and Strick, 2004). Reciprocal projections concerning the striatum and GPi/SNr are divided into two distinct pathways, namely a “direct” monosynaptic connection, and an “indirect” projection via the interpolated GPe/STN. GPi/SNr output projects mainly to ventral anterior (VA) and ventrolateral (VL) thalamic nuclei, which project to the cerebral cortex. Minor BG projections extend to the intralaminar centromedian and parafascicular thalamic nuclei, and brainstem structures such as the superior colliculus (SC), PPN, and reticular formation. The striatum also obtains prominent dopaminergic SNc input (Galvan and Wichmann, 2008). The BG are a major brain system modulated by dopaminergic input from the SN (Albin et al., 1989) with profound effects on behavior.

**Figure 2:**
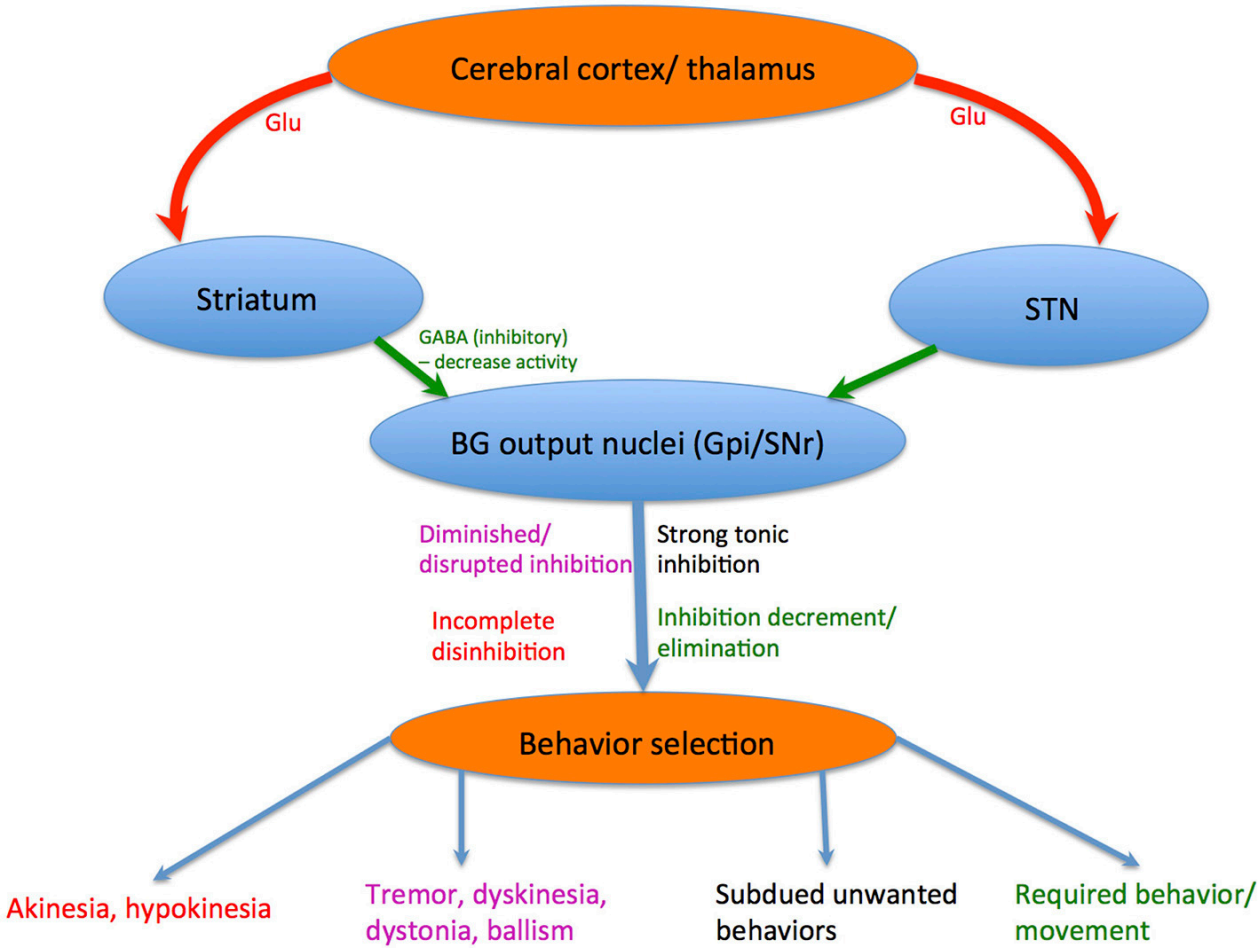
Behavior selection: Red lines depict glutamatergic pathways, whereas green lines depict GABAergic pathways. Blue lines depict chemically composite pathways. The corresponding colored notations show how each different movement disorder is elicited.

The striatum and STN obtains glutamatergic afferents from exclusive cerebral or thalamic regions and transfer this information to BG output nuclei, namely the GPi and SNr. These BG output nuclei fire tonically and rapidly (DeLong and Georgopoulos, 2011), thus brain areas receiving inputs from the BG are continuously under strong tonic inhibitions (Hikosaka, 2007). Decreases in SNr/ GPi neuronal activity is caused by direct input from the neostriatum, which are also GABAergic and inhibitory (Yoshida and Precht, 1971; Hikosaka et al., 1993b). An appealing theory states that the BG's chief purpose is apt behavior selection (Hikosaka et al., 1993a; Mink, 1996; Nambu et al., 2002), where unwanted behaviors are subdued by SNr/GPi-induced inhibition preservation or increment whilst required behaviors are liberated by SNr/GPi-induced inhibition decrement or elimination. Patients with BG dysfunctions portray involuntary movement disorders such as tremor, dyskinesia, dystonia, chorea, athetosis, and ballism (Denny-Brown, 1968). These involuntary movements are instigated by a disruption of the SNr/GPi-induced inhibition, consistent with parkinsonian symptoms displaying involuntary tremulous movements, diminished muscular power whether in activation or not, impaired posture with an inclination to bend the trunk forwards, festination from walking to running or poverty and slowness of movement without paralysis (DeLong and Wichmann, 2007), where the senses and intellect are uninjured initially. However, these patients also display difficulty in initiating purposeful movements known as akinesia, or slow and small movements known as bradykinesia and hypokinesia (see Figure 2). This movement disorder is elicited by an incomplete disinhibition of the SNr/GPi-induced inhibition on thalamocortical systems (Burbaud et al., 1998; Stein, 2009), ensuing in gait disturbances with difficulties initiating or terminating walking (Azulay et al., 2002). Additionally, dyskinesias induced by the DA pre-cursor levodopa (L-DOPA) or DA agonist apomorphine, are concomitant with the inadequate suppression of BG GABAergic output (Nevet et al., 2004). This leads to abnormal oscillatory firing of motor neurons in the aforementioned areas, inducing tremor or other involuntary movements.

The BG is known for controlling locomotion and posture via SNr-GABAergic output (Takakusaki et al., 2004c). In PD patients, GABAergic BG output levels are abnormally increased (Miller and DeLong, 1987; DeLong, 1990; Filion, 1991). Takakusaki et al. (2004b) proposed that gait disturbances in PD are produced by abnormal increases in SNr-induced inhibition of the MLR. Furthermore, muscle rigidity might result from abnormally increased PPN inhibition that would otherwise produce muscle relaxation (see Figure 1B). Dystonia could be triggered by BG GABAergic output diminution to the PPN, depicted by focal and involuntary muscle tone, posture, or movement changes (Starr et al., 2005). Augmented GABAergic outputs would thus overwhelm target areas including the SC, MLR, PPN, thalamo-cortical circuits, and feasibly mouth movement and vocalization centers, ensuing in akinesia or hypokinesia (see Figure 2).

Another reason for a deranged BG-GABAergic output in the SNr/GPi would result from inputs coming from the GPe/neostriatum (Hikosaka, 2007). These motor features are often accompanied by non-motor issues such as depression, anxiety, autonomic dysfunction, sleep disorders, and cognitive impairment as a result of DA deficiency in non-motor portions of the striatum and more widespread progressive pathologic changes in the brainstem, thalamus, and eventually, the cerebral cortex (Braak et al., 2003).

## The PPN and BG-brainstem system

Ascending PPN projections provide substantial innervation to the SNc, STN, and GP (Mehler and Nauta, 1974; Graybiel, 1977; Nomura et al., 1980; Saper and Loewy, 1982; Edley and Graybiel, 1983; Jacobs and Azmitia, 1992; Spann and Grofova, 1992; Lavoie and Parent, 1994a,b,c). The inconsistency between the number of ascending and descending projections indicate that the PPN is not a major output component, but a modulating structure as it is part of the many auxiliary loops in BG circuitry and activity. This is because of its strategic position and network with the MCx, thalamus, SN, STN, and CuN. PPN neurons exert excitatory action upon various BG components facilitated mainly by Ach (Woolf and Butcher, 1986). However, the presence of glutamate and various neuropeptides within (Clements and Grant, 1990; Clements et al., 1991; Côté and Parent, 1992) suggest that the PPN also applies an expansive range of effects upon BG neurons through various chemo-specific neuronal systems (Parent and Hazrati, 1995). PPN neurons directly influence BG output nuclei, namely the SNr and GPi, and therefore directly affect information processed within the BG before approaching targets such as the thalamus. Since the PPN establishes highly reciprocal connections with the BG than any other brain region, both these structures exhibit complex physiological interdependence crucial for physiologic function (Mena-Segovia et al., 2004, 2008b). These structures are interconnected either directly or indirectly with every element, and the BG receives large converging input from the PPN (Garcia-Rill, 1991; Pahapill and Lozano, 2000; Mena-Segovia et al., 2004; Alderson and Winn, 2005).

**Figure 3:**
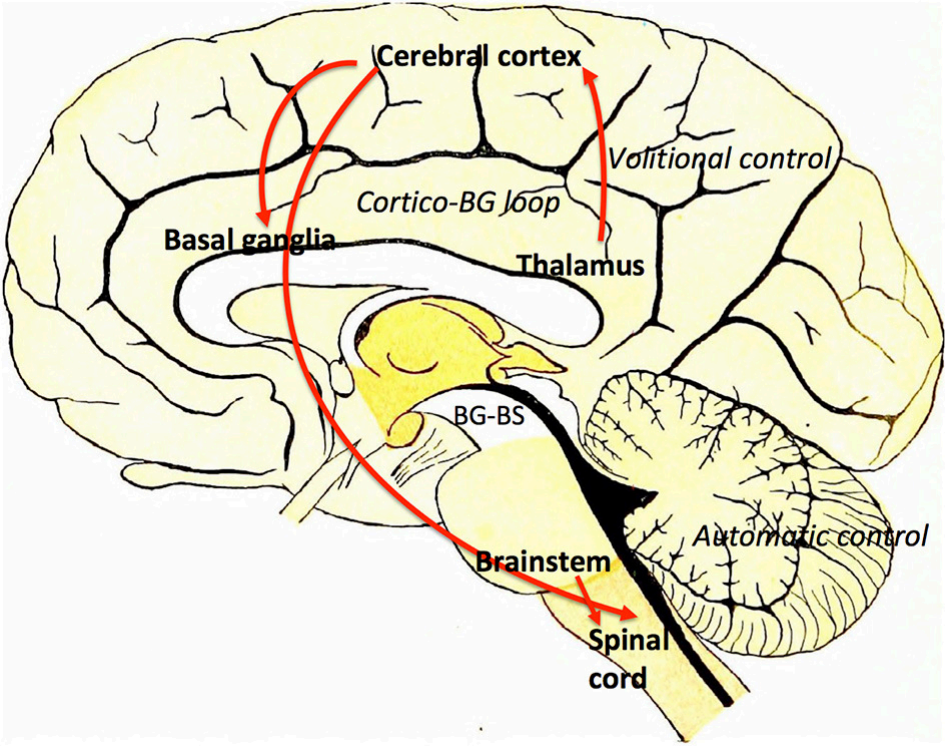
Volitional and automatic control of locomotor movements by the BG-BS system: GABAergic BG output to thalamocortical and brainstem neurons assimilates volitional and automatic movement control processes. Adapted from Villiger and Piersol (1912).

The BG–brainstem (BG–BS) system functions throughout the mesopontine tegmentum in controlling diverse behavioral expressions. This includes automatic movement control comprising periodic limb movements and postural muscle tone adjustments throughout locomotion integrated with voluntary control. It is also involved in awake–sleep state regulation. The BG-BS system is thus accountable for the manifestation of volitionally-directed and emotionally-instigated motor behavior consolidation, and dysfunction of this system together with the cortico-BG loop triggers behavioral disorders (Takakusaki et al., 2004c). The BG performs planning and implementation of deliberate movements through parallel BG-thalamocortical loop sequences (Alexander and Crutcher, 1990; DeLong, 1990; Turner and Anderson, 1997), directing outflow to motor networks in the brainstem (Inglis and Winn, 1995; Hikosaka et al., 2000; Takakusaki et al., 2003) where central neuronal complexes for muscle tone and locomotor movement control are located (Garcia-Rill, 1991). Thus, BG outputs project through thalamocortical loops to the brainstem, and are involved in postural muscle tone and locomotion integrative assimilation (Takakusaki et al., 2004b). Hikosaka et al. (2000) postulates that the BG utilizes two types of output to regulate movements; one via thalamocortical systems, and another via brainstem motor networks (see Figure 3).

BG output to the cerebral cortex regulates voluntary movement control processes, whereas specific movement patterns such as saccades (Hikosaka and Wurtz, 1983a,b,c; Hikosaka, 1991; Hikosaka et al., 2000; Sparks, 2002), vocalization (Düsterhöft et al., 2000), and locomotion (Rossignol, 1996) are generated by exclusive neuronal systems in the brainstem and spinal cord. MCx projections are directed to the PPN (Matsumura et al., 2000) and pontomedullary reticular formation (Matsuyama and Drew, 1997), where muscle tone regulation and the locomotor system are coordinated simultaneously by dual feedback via net BG inhibition and MCx excitation to the brainstem. In PD, GABAergic BG output is overactive (Wichmann and DeLong, 1996, 2003), ensuing in sluggishness and movement decreases by thalamocortical neurons, known as bradykinesia and hypokinesia respectively. Contrarily, increases in BG inhibition together with PPN cortical excitation reductions would increase the level of muscle tone, known as hypertonus. Likewise, excessive MLR inhibitions and cortical excitation decreases in the brainstem reticular formation would educe gait failure. Furthermore, primary MCx inactivity would disrupt the locomotor programming necessary for defined gait control (Hanakawa et al., 1999; Pahapill and Lozano, 2000). Resultantly, this would constrain the degree of freedom for locomotion (Takakusaki et al., 2004c). Gait disturbances where delays are seen in freezing of gait (FoG), stance phase increases in movement sequences and movement speed decreases are also seen in PD invalids (Morris et al., 1994; Pahapill and Lozano, 2000). BG–BS system impairment would be the principal foundation for PD-induced gait deficiencies (Takakusaki et al., 2004c) as these gait failures resemble SNr-stimulated movement (Takakusaki et al., 2003).

**Figure 4:**
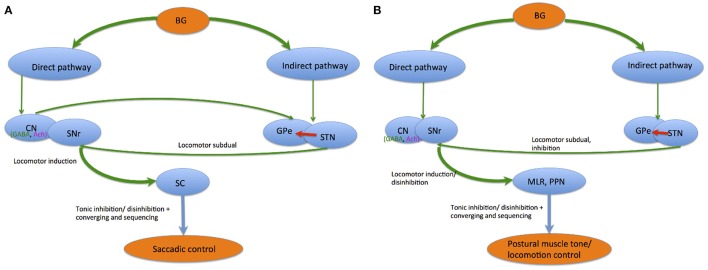
Saccadic control **(A)** and postural muscle tone/ locomotion control **(B)** by the direct and indirect pathways.

In saccadic control, the direct and indirect pathways within the BG (Alexander and Crutcher, 1990; DeLong, 1990) cause GABAergic SNr tonic neuronal inhibition of SC output neurons, consequently preventing unnecessary saccades. The direct pathway from the CN to SNr results in SC neuronal disinhibition by eradicating this constant inhibition. Specifically, phasic GABAergic output neuronal activity in the CN permits saccade occurrence via tonic SNr-SC inhibition suspension (Hikosaka, 1989). The indirect pathway, involving the GPe and STN, further enhances the SNr-SC inhibition via excitatory cortical input (Nambu et al., 2002). Hence, direct CN-SNr and indirect CN-GPe-STN-SNr pathways induce contrasting SNr-SC system effects. Concurrent interactions within the two pathways generate additional discriminating information and heighten the target systems' neural signal spatial contrast. Inversely, behavior interchange from locomotor subdual when the indirect pathway dominates, to locomotor induction when the direct pathway dominates, is produced via consecutive communication of the pathways. This effect enhances temporal contrast. Thus, BG saccadic control can be summarized via two mechanisms. The first is by enhancement of tonic inhibition and disinhibition, while the second mechanism is through converging and sequencing. These two modules are elicited via direct and indirect pathway communication, and might influence brainstem networks besides thalamocortical networks (Hikosaka et al., 2000) (see Figure 4).

Similarly, disinhibition and inhibition regulations are key mechanisms for BG postural muscle tone and locomotion control. Locomotor and muscle tone control systems are normalized by the direct and indirect pathway balance via muscle tone inhibitory regions in the PPN, MLR, and SC receiving GABAergic input from the SNr. During locomotor movement preparation, tonic SNr neuronal tonic activity continuously inhibits both systems. When an activating signal occurs, the direct pathway releases activity in these systems, causing locomotion initiation followed by muscle tone level reduction. Parallel SNr organization to the MLR/PPN also regulates muscle tone level accompanying the initiation and termination of locomotion (Takakusaki et al., 2004c).

Cholinergic PPN neuronal loss in PD (Hirsch et al., 1987; Zweig et al., 1987, 1989; Jellinger, 1988) also attributes to attentive and cognitive damages and sleep defects (Scarnati and Florio, 1997). This validates that the BG-BS are also involved in non-motor function, specifically in REMS regulation, arousal and emotional motor behaviors (Takakusaki et al., 2004c).

### Gait and locomotion

**Figure 5:**
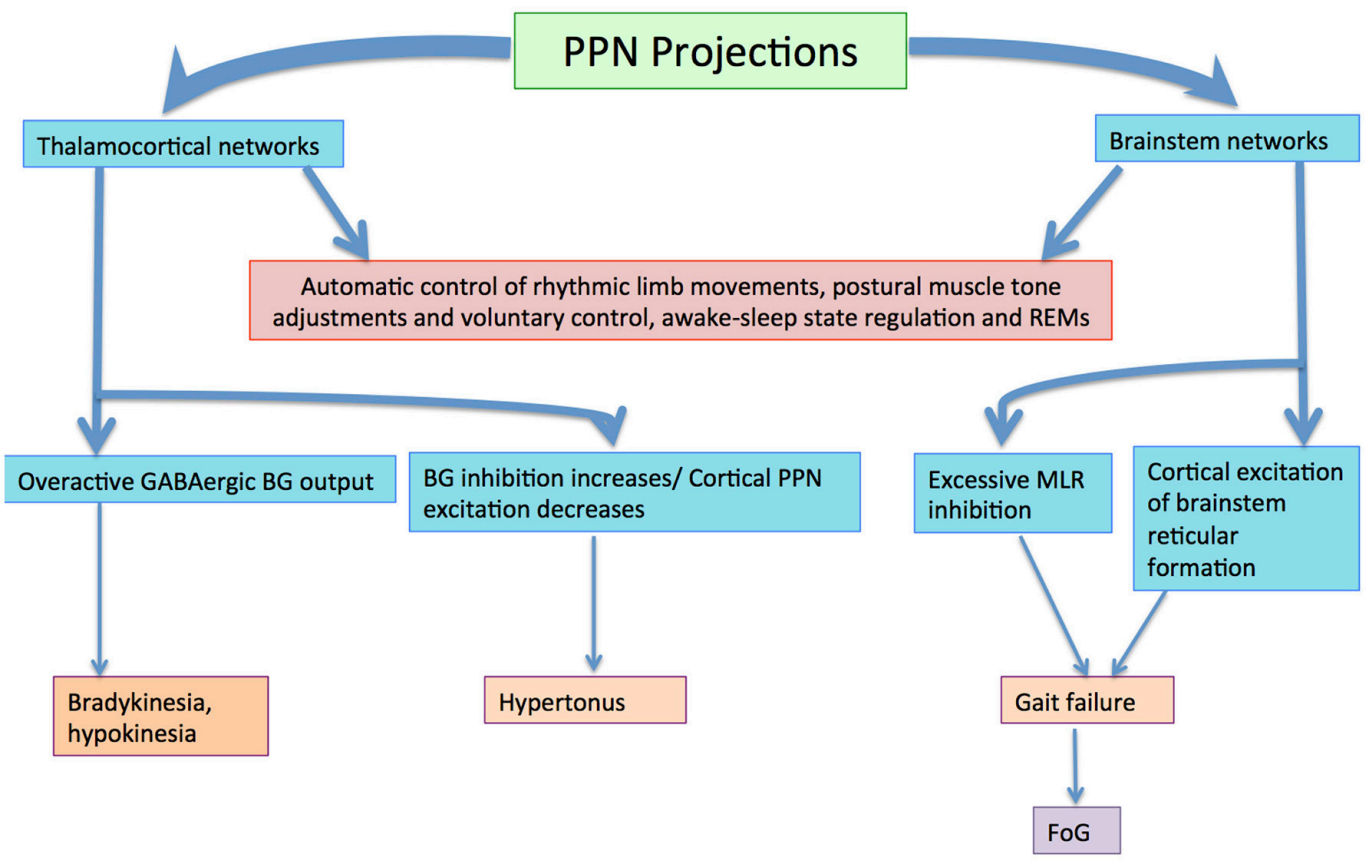
The involvement of the PPN in the neuropathology of PD.

As mentioned, the PPN is a central part of the MLR within the brainstem, where it generates and supports lower controlled locomotion (Skinner and Garcia-Rill, 1984; Skinner et al., 1990) via descending projections innervating foci in the lower brainstem and medulla, comprising the oral pontine reticular nucleus, the GiN, the medioventral medulla, and spinal cord regions (Mitani et al., 1988; Rye et al., 1988; Nakamura et al., 1989; Semba et al., 1990; Grofova and Keane, 1991; Scarnati et al., 2011). These projections are associated with gait control and posture primarily via locomotion inhibition, where increasing levels of high stimulation drives the frequency of stepping from walking to running (see Figure 5) (Garcia-Rill et al., 1987; Garcia-Rill, 1991).

The cholinergic PPNc induces locomotion (Garcia-Rill et al., 1987) together with other brainstem regions via prominent sensory nuclei stimulating locomotion through direct outputs to spinal cord regions of recognized locomotor generators (Pahapill and Lozano, 2000). PPN neuronal response to somatosensory excitation (Grunwerg et al., 1992; Reese et al., 1995) combined with cholinergic PPN neuronal thalamic projections and inputs from lamina 1 of the spinal cord, advocates that the PPN modulates sensory information to thalamic nuclei. Thus, the PPN plays a role as a dispatch amid the cerebral cortex and spinal cord, providing feedback information vital for posture and gait initiation modulation. This is enabled by ascending thalamic cholinergic projections and deep cerebellar nuclei networks (Pahapill and Lozano, 2000).

Non-cholinergic PPNd neurons receive input from the BG and limbic structures, propositioning that the PPN acts as an assimilator for BG motor choice output and incentive-motivated commands from the striatal-pallidal complex to deliver motivationally influenced activation of motor pattern generators in the pons, medulla and spinal cord (Inglis and Winn, 1995). Such factors affect motor function like kinesia paradoxica. Treatment via PPN activation would improve motor planning and permit increasing motivational ability in stimulating preserved motor programs for stereotyped movements (Pahapill and Lozano, 2000).

### Reward, motivation, and compulsion

The PPN is accountable for the phasic activity bursts in SNc DA cells, which plays a key role in learning and preserving instrumental tasks (Scarnati et al., 1988; Futami et al., 1995). Primary PPN reward stimuli originate from the lateral hypothalamus, but excitatory reward-prediction stimuli spawns a condition stimulus-elicited DA surge traversing the ventral striatum–pallidum pathway, receiving predominantly limbic cortex input (Schultz et al., 1992). Striosomal cells regulate response to primary reward after conditioning via suppressing DA burst response through the striosomal-SNc pathway (Gerfen, 1992). Striosomal cells also modulate the adaptive scheduled reward expectation that annuls the predicted reward at the predicted interval (Schultz et al., 1997). DA cell activity is therefore an exclusive parallel act of PPN inactivation, compared to a secondary influence on motivation or abridged capability of task performance. The PPN thus serves as an integrative interface amidst innumerable stimuli necessary for executing intended behaviors (Kobayashi et al., 2004). This enables the fundamental activities for motor command initiation and external sensory dispensation via arousal regulation and attentive conditions through dopaminergic systems (Takakusaki et al., 2004c).

PPN lesions ensued in impaired attention (Inglis et al., 2001) and memory learning during a trained incitement and a prime reward (Inglis et al., 1994, 2000). This advocates that PPN inactivation has variable effects on non-dopaminergic cells in the VTA. Firstly, PPN neurons respond to the same task stimuli, whether visual- or auditory-activating DA cells. Secondly, PPN responses are governed by phasic inception patterns observed in DA cells. Thirdly, PPN cells respond before DA cells, permitting PPN-DA information transfer. Finally, PPN suppression subdues DA cell responses to stimuli without upsetting baseline firing frequency (Pan and Hyland, 2005). Therefore, auditory, visual, and somatosensory trained incitements stimulate DA cells (Romo and Schultz, 1990; Schultz and Romo, 1990), with bias toward auditory incitements at tremendously brief latencies, strictly programmed to the stimulus interval. The preference of PPN cells for tone and light increases the likelihood that homogenous afferent projections regulate DA cell action, with biasness for either constituent (Wallace and Fredens, 1988; Comoli et al., 2003). Thus, the PPN and SC supplement each other in dispatching sensory knowledge of different stimuli. PPN neuronal activity predisposed toward auditory responses has a functionally imperative role in reducing DA cell responses without substantial effects on baseline firing rate via a visual component inactivation. This establishes that the PPN selectively controls DA cell bursting rather than tonic resting activity (Floresco et al., 2003), and that PPN inputs are necessary for producing DA cell surge reactions to significant sensory stimuli (Schultz, 1998; Brown et al., 1999). The PPN is therefore imperative for arousal, attention, motivation, learning, and specifically stimulated–reward conditions (Steckler et al., 1994).

PPN lesions do not disrupt brain stimulation reward value however (Waraczynski and Perkins, 1998), suggesting that it performs as a primary sensory and motivational system interface toward delivering communication signals irrespective of reward value. DA cells typically react staggeringly when signals are reward-connected, while PPN cells react non-contingently, suggesting that separate, reward-information-bearing pathways gate PPN inputs. Hence PPN inputs have a dual role, to provide precise and brief latent information toward sensory stimuli intervals and advanced-level function information transmission concerning signals dispatched via sensory-attention regulating mechanisms (Pan and Hyland, 2005).

Research establishes that lateral hypothalamic brain stimulation not only rewards, but also drive-induces (Coons et al., 1965; Glickman and Schiff, 1967). Rewarding hypothalamic brain stimulation depends on trans-synaptic induced release of Ach in the VTA (Yeomans et al., 1993), where dominant portions of these fibers synapse in PPN cholinergic efferents relaying messages back to the VTA (Yeomans et al., 1993). These cholinergic PPN neurons provide non-specific facilitation for reward-connecting behaviors, and therefore act as a relay amid limbic-incentive organization and brainstem locomotor machinery (Steckler et al., 1994). Due to its position within the mesolimbic DA system encompassing the VTA and NAcc, it is entangled in brain mechanisms and neural circuitry formation involved in reward processing, which can lead to motivation and compulsion.

### Rapid eye movement sleep (REMS)

PPN and LDT cholinergic neurons are involved in arousal state maintenance and REMS generation (Rye, 1997). During sleep, PPN cholinergic activation of the cortex transpires via projections to the thalamocortical network to subdue slow delta waves and elicit cortical stimulation (Belaid et al., 2014). This leads to REMS, through REM-on and –off cellular activity together with the locus coeruleus and dorsal raphe nuclei (DRN) (McCarley and Chokroverty, 1994). Reduced inhibitory input from the SNr/GPi nuclei to the PPN results in higher intrinsic activity causing cortical activation and electroencephalography (EEG) desynchronization (Reiner et al., 1988) leading to REMS (Steriade, 1996). These neuronal mechanisms that induce REMS and muscular atonia together with PPN cholinergic projections are under SNr GABAergic inhibition regulation.

PD patients are known to experience several sleep disorders, including reduction of REMS sleep period and REMS behavior disorder (RBD) (Bliwise et al., 2000; Eisensehr et al., 2001). This is because decreases in BG dopaminergic activities is also involved in REMS reduction and RBD (Rye et al., 1999; Albin et al., 2000), hence providing a lucid explanation for the pathogenesis of sleep disturbances in PD (Takakusaki et al., 2004c; French and Muthusamy, 2016). A summary of the different maladies associated with the PPN in PD are listed in Table 1.

**Table 1:** Maladies associated with the PPN in PD, the source and affected brain components, as well as its consequence/ indications.

**Malady type**	**Source**	**Brain components affected**	**Consequence/ Indications**
**LOCOMOTOR**			
Akinesia/bradykinesia/hypokinesia	Overactive GABAergic BG output	MCx, thalamus, BG, PPN, SC, MLR	Decreases in velocity and amount of movement
Hypertonus	Increases in BG inhibition Increases in PPN inhibition Decreases in cortical PPN excitation Direct-indirect pathway imbalance via increased SNr inhibition	MCx, BG, PPN	Increased muscle tone
Dystonia	Diminution or instability of BG GABAergic outputs to the PPN	BG (SNr, Gpi), PPN	Central and spontaneous fluctuations in muscle tone, posture, or locomotion
Gait disturbances/ failure	Excessive MLR inhibition via SNr-GABAergic output Cortical excitation of brainstem reticular formation Decreases in cortical stimulation of the brainstem reticular formation Inactivity of the PMC Dysfunction of BG-BS system Direct-indirect pathway imbalance	MLR MCx, brainstem Primary MCx SNr, PPN, MLR, SC	Uncontrolled gait Limited movement Freezing of gait, increases in stance phases in locomotor cycles and decreases in locomotor velocity
**COGNITIVE**			
Impaired attention and memory learning	PPN lesions	PPN	Inability to concentrate, unable to retain memory
Motivation and compulsion	PPN lesions, impaired brain mechanisms and neural circuitry formation involved in reward processing	NAcc, VTA, PPN	Obsessive compulsive disorder
**SLEEP**			
REMS	Decreases in BG dopaminergic activity	SNr, GPi, DRN, LDT, PPN	REMS behavior disorder

## Treatment/deep brain stimulation

Electrode recordings in deep brain stimulation (DBS) postulate that uncontrolled abnormal pathological oscillations throughout motor networks in the STN, GP, and thalamus are concomitant with motor symptoms in PD (Hammond et al., 2007). Similarly BG networks oscillating at a pathological beta (β) range of 20 Hz, driven by cerebral neurons firing in either “burst” or “tonic” modes is associated with akinesia (Stein, 2009). Successful alleviation of akinesia with L-DOPA enables the system to break away from this pathological beta repression (Kühn et al., 2008). High frequency stimulation, applied via DBS also subdues pathological synchronization (Brown and Eusebio, 2008). PD symptoms are lessened by DBS via preventing pathological neuronal network oscillations that destabilize them, and are successful as they abolish nodes responsible for oscillation generation itself. DBS is thus permanently effective by driving neurons tonically so that pathological oscillations causing the burst/silence mode are reversible.

Neurophysiologically, cortical bursts normally govern PPN input and orchestrate field potentials and neuronal discharges to the cortical rhythm so that PPN local field potentials and neuronal discharges are synchronized with those of MCx activity (Aravamuthan et al., 2008). However, these synchronies reverse after cell lesions and PPN neurons fire mostly throughout positive swings in the cortex when they should be silent. This indicates that inhibitory GP and SNr output is predominant input to the PPN, rather than excitatory MCx output. Stimulation of the GP and SNr also requires adequate glutamate conduction. This substitutes dominant PPN firing via inhibitory input for normal excitatory input from the MCx and STN to the PPN. The β-band is responsible for associated akinesia, where β suppression increases with the complexity of the intended movement while its latency predicts movement onset. This means that the earlier the suppression, the shorter the movement latency. Therapeutic interventions reducing akinetic symptoms reduce enhanced synchronization in the β band and facilitates regular gamma oscillations (Brown et al., 2001).

The PPN area might be a good prospective DBS objective concerning freezing and other gait disorders' treatment associated with PD, where data shows that cholinergic denervation due to PPN neuronal degeneration causes DA non-responsive gait and balance impairment (Karachi et al., 2010; Grabli et al., 2013). Imaging studies in PD patients propose that unilateral PPN DBS intensifies cerebral blood flow bilaterally into the central thalamus and cerebellum (Ballanger et al., 2009). However, recognized assessments support bilateral DBS (Khan et al., 2012) ascertained to be superior particularly for controlling FoG. Thevathasan et al. (2012) further supports this, concluding that bilateral stimulation was more successful in a specific subgroup of PD patients by ~200%. This study exhibited concrete unprejudiced, double-blinded proof that an explicit subcategory of Parkinsonian patients benefit from bilateral stimulation of a caudal PPN region just below the pontomesenphalic junction that discriminately improves FoG. This did not include inconsistencies in step length however, which could be furtive freezing interrupting the smooth execution of gait (Thevathasan et al., 2012). Long-term outcomes would unquestionably need further substantiation via supplementary studies or randomized trials with longer follow-up periods involving a higher number of patients and exclusive criteria.

Most PPN DBS studies denote alleviation in patients disturbed by freezing and falls although outcomes are variable. This possibly reflects patient choice, target option heterogeneity, surgical procedure differences and stimulation protocols (Hamani et al., 2016a). This leads to a number of challenges to be solved, including the prime target identification, surgical method choice that optimizes electrode placement, precision, and impact of surgical procedures, intraoperative target reliability, and procedural modifications in postoperative electrode position validation. Nonetheless, the procedure appears to provide benefit to selected patients and is comparatively safe. One important limitation in comparing studies from different centers and analyzing outcomes is great target variability and surgical techniques (Hamani et al., 2016b).

Chronic PPN stimulation is usually combined with stimulation of other targets, including the STN, GPi, and the caudal zona incerta due to its superiority compared to PPN DBS alone. However, combined stimulation poses challenges in the effectual assessment of DBS in each target, and also in enlightening the complex relationship between medication and stimulation. A particular problem is the use of low-frequency PPN stimulation and high-frequency stimulation in other targets, where this necessitates intricate programming or utilization of a supplementary pulse generator. Another issue is the concordance on the ideal target position within the PPN region, where it is ambiguous as to whether electrodes should be implanted in the rostral PPN at the level of the inferior colliculus or caudal PPN in a region about 4 mm below the inferior colliculus. A reasonable approach would be to insert contacts in both rostral and caudal PPN regions since available data is still vague, and also because the PPN is oriented along the long axis of the brainstem. Since the PPN is partially degenerated in PD, smaller-spaced electrodes might be preferable. It would also be vital to develop a specified set of resting and movement-related intraoperative local field potentials while conducting PPN DBS as frequency bands in the alpha, β, and theta ranges and movement-related potential were all recorded from the PPN region (Hamani et al., 2016b).

PPN DBS is still a relatively novel intervention in PD, and the numerous challenges mentioned before must be resolved. Despite these trials, the procedure provides benefit to selected patients and appears relatively safe. The future role of PPN DBS in the armamentarium of surgery for PD patients is still uncertain. Unquestionably, more studies are needed to provide more solid data on the advantages and limits of chronic stimulation (Hamani et al., 2016b).

## Conclusion

Understanding the function of the PPN and its utility in the many neuronal circuitries of the brain is vital for neurophysiological knowledge. This would ensue in the understanding of how maladies such as Parkinson's disease occurs along amid its consequences, and subsequently help in producing the appropriate treatment needed to cure and control these disorders. A promising and long-term treatment would be DBS, which could vastly improve patients' quality of life. Further studies would definitely need to be conducted to elucidate further on such disorders especially in terms of genetics and biochemistry.

## Author contributions

IF was the main author of this paper who wrote this manuscript as part of a research project in order to understand better and summarize the physiology and pathophysiology of the pedunculopontine nucleus in associated with Parkinson's disease. KM provided the main oversight and general guidance in the completion of this manuscript.

### Conflict of interest statement

The authors declare that the research was conducted in the absence of any commercial or financial relationships that could be construed as a potential conflict of interest.

## Abbreviations

Ach: Acetylcholine
ARAS: Ascending reticular activating system
BG: Basal ganglia
BG-BS: Basal ganglia-brainstem
CN: caudate nucleus
CuN: Cuneiform nucleus
DA: Dopamine
DBS: Deep brain stimulation
GABA: Gamma aminobutyric acid
GiN: Gigantocellular reticular nucleus
GP: Globus pallidus
GPe: Globus pallidus externa
GPi: globus pallidus interna
LN: Lewy neurites
LB: Lewy bodies
LDT: Laterodorsal tegmental nucleus
MCx: Motor cortex
MLR: mesenphalic locomotor region
NAcc: Nucleus accumbens
PPN: Pedunculopontine nucleus
PPNc: Pedunculopontine nucleus pars compacta
PPNd: Pedunculopontine nucleus pars dissipatus
PD: Parkinson's disease
REMS: Rapid eye movement sleep
VTA: Ventral tegmental area
VA: ventral anterior nucleus
VL: ventrolateral nucleus
SC: Superior colliculus
SN: Substantia nigra
SNc: Substantia nigra pars compacta
SNr: Substantia nigra pars reticulate
STN: Subthalamic nucleus.
